# Ghost-Free HDR Imaging in Dynamic Scenes via High–Low-Frequency Decomposition †

**DOI:** 10.3390/s25227013

**Published:** 2025-11-17

**Authors:** Xiang Zhang, Genggeng Chen, Fan Zhang, Yongzhong Zhang

**Affiliations:** 1 College of Information and Control Engineering, Xi’an University of Architecture and Technology, Xi’an 710055, China; chengeng0613@xauat.edu.cn (G.C.);; 2 China United Network Communications Group Co., Ltd., Shaanxi Branch, Xi’an 710000, China

**Keywords:** high dynamic range imaging, ghost-free HDR, High-Low Frequency Decomposition

## Abstract

Generating high-quality high-dynamic-range (HDR) images in dynamic scenes remains a challenging task. Recently, Transformers have been introduced into HDR imaging and have demonstrated superior performance over traditional convolutional neural networks (CNNs) in handling large-scale motion. However, due to the low-pass filtering nature of self-attention, Transformers tend to weaken the capture of high-frequency information, which impairs the recovery of structural details. In addition, their high computational complexity limits practical applications. To address these issues, we propose HL-HDR, a high–low-frequency-aware ghost-free HDR reconstruction network for dynamic scenes. By decomposing features into high- and low-frequency components, HL-HDR effectively overcomes the limitations of existing Transformer and CNN-based methods. The Frequency Alignment Module (FAM) captures large-scale motion in the low-frequency branch while refining local details in the high-frequency branch. The Frequency Decomposition Processing Block (FDPB) fuses local high-frequency details and global low-frequency context, enabling precise HDR reconstruction. Extensive experiments on five public HDR datasets demonstrate that HL-HDR consistently outperforms state-of-the-art methods in both quantitative metrics and qualitative evaluation.

## 1. Introduction

Contemporary digital cameras are limited by the capabilities of their sensors, making it difficult to capture the full dynamic range of real-world scenes. In contrast, High-Dynamic-Range (HDR) imaging can encompass a much wider range of light intensities, providing a more accurate representation of real-world luminance distributions. HDR imaging has become a key technique in modern visual applications, capable of faithfully reproducing lighting in complex scenes that contain both extremely bright and dark regions. Typical applications include photography and cinematography, where HDR enhances tonal depth and detail representation; virtual reality and game rendering, where it improves lighting realism and immersive experience; and autonomous driving and intelligent surveillance, where it helps maintain clear visibility of critical targets under challenging illumination conditions.

There are various approaches to generating HDR images, among which one of the most common methods is to reconstruct an HDR image by fusing multiple Low-Dynamic-Range (LDR) images captured under different exposure settings. However, during multi-exposure HDR reconstruction, object motion or camera shake may cause temporal inconsistencies or information loss due to overexposure, resulting in ghosting artifacts. This phenomenon remains one of the major challenges in multi-exposure HDR imaging.

To tackle the challenges associated with ghosting in HDR imaging, various methodologies have been developed. Traditional techniques commonly employ methods such as alignment-based methods [1,2], rejection-based methods [3,4,5], and patch-based methods [6,7] to eliminate or align motion regions in images. However, the efficacy of these methods is largely contingent upon the performance of preprocessing techniques, such as optical flow and motion detection. And when dealing with significant scene motion, the results of these methods typically turn out to be rather unsatisfactory. With the advancement of Deep Neural Networks (DNN), several CNN-based methods [8,9,10,11,12] have been applied in ghost-free HDR imaging. Among them, the “alignment-fusion” paradigm has shown remarkable success, especially in scenarios involving large-scale motion. Moreover, Transformer-based approaches [13,14], which can capture long-distance dependencies, are introduced as an alternative to CNNs. These methods further enhance HDR imaging performance and are adopted by the current mainstream state-of-the-art methods. However, Transformers still face two major challenges in ghost-free HDR imaging. On one hand, local details and global information are crucial for restoring multi-frame HDR content, while the self-attention mechanism of pure Transformers often exhibits a low-pass filtering effect, reducing the variance of input features and overly smoothing patch tokens. This occurs because self-attention essentially averages features across different patches, suppressing high-frequency information that is vital for distinguishing fine structural details, thereby limiting the Transformer’s ability to capture high-frequency local details [13]. On the other hand, HDR images are typically high-resolution, and the computational complexity of self-attention grows quadratically with the spatial dimensions of the input feature map. This results in significant computational and memory overhead in high-resolution scenarios, restricting the practical application and scalability of Transformers in high-resolution HDR imaging tasks.

Considering that the high- and low-frequency components of an image correspond to local details and global structures, respectively, we propose a frequency-decomposition-based ghost-free HDR image reconstruction network. In both the cross-frame alignment and feature fusion stages, features are decomposed into high- and low-frequency components and processed according to their respective characteristics. Since high-frequency components represent local structures while low-frequency components characterize global information, we leverage the low-pass filtering property of average pooling (AvgPool) to decouple features into high-resolution high-frequency components and low-resolution low-frequency components.

Specifically, global motion or long-range dependencies can be effectively represented by low-frequency features without requiring high-resolution feature maps, while high-frequency features focus on fine-grained local structures that need high-resolution maps and are better modeled by local operators. Based on this, we adopt a dual-branch architecture in both stages to balance global information and local details.

In the cross-frame alignment stage, we propose the Frequency Alignment Module (FAM). The low-frequency branch employs a lightweight UNet to learn optical flow and align non-reference frames to the reference frame, efficiently capturing large-scale motion while reducing computational cost. Meanwhile, the high-frequency branch combines convolution and attention to adaptively refine edges and textures, suppressing ghosting and preserving structural consistency.

In the feature fusion stage, we design the Frequency Decomposition Processing Block (FDPB). The high-frequency branch uses a Local Feature Extractor (LFE) to capture details and enhance cross-frame high-frequency information, while the low-frequency branch adopts a Global Feature Extractor (GFE) to model long-range dependencies. To alleviate information loss caused by downsampling, we further introduce a Cross-Scale Fusion Module (CSFM) for effective cross-resolution integration.

By integrating FAM and FDPB, we propose the High–Low-Frequency-Aware HDR Network (HL-HDR), which consists of two stages: cross-frame alignment and feature fusion. FAM enables accurate motion modeling and detail preservation, while FDPB hierarchically captures both global and local contexts, leading to high-quality, ghost-free HDR reconstruction.

The main contributions are summarized as follows:

- We propose a novel alignment method, FAM, in which the low-frequency branch captures large-scale motion through optical flow alignment, while the high-frequency branch refines local edges and textures, effectively suppressing ghosting.
- The FDPB module, introduced in our work, addresses low-frequency components by employing a multi-scale feature extraction approach in conjunction with Transformer mechanisms to collectively capture global information. For high-frequency components, we employ small convolutional kernels and densely connected residual links to effectively extract local feature information. This strategic design in our model achieves a harmonious balance between speed and precision.
- A plethora of experiments have substantiated that the proposed methodology, denoted as method HL-HDR, attains state-of-the-art (SOTA) performance in HDR imaging tasks. Furthermore, it yields visually appealing outcomes that align with human perceptual aesthetics.

This work is an extended version of our conference paper [15] presented at the International Joint Conference on Neural Networks (IJCNN 2024). Compared with the conference version, it incorporates a substantial amount of new material. (1) To address the issue of ghosting in moving regions, we optimized the cross-frame alignment stage by designing the FAM module: the low-frequency branch aligns features using optical flow, while the high-frequency branch adaptively refines local edges and textures through convolution and attention mechanisms, effectively suppressing ghost artifacts and maintaining structural consistency. (2) We conducted comparative experiments on additional datasets and against the latest methods, fully demonstrating the advantages of our improved approach. (3) The ablation studies are more detailed and clear, thoroughly verifying and analyzing the contributions of each module.

## 2. Related Work

Presently, HDR deghosting techniques can be primarily classified into alignment-based methods, rejection-based methods, patch-based methods, and CNN-based methods.

### 2.1. HDR Deghosting Methods

**Alignment-based Method.** These methods aim to register all LDR images to a reference image using either rigid or non-rigid algorithms. Bogoni [1] utilized optical flow to estimate motion vectors, while Pece and Kautz [5] computed the Median Threshold Bitmap (MTB) for input images to detect regions of motion. Kang et al. [16] transformed the intensities of LDR images into the luminance domain by leveraging exposure time information and computed optical flow to identify corresponding pixels among the LDR images. However, both rigid and non-rigid alignment methods exhibit susceptibility to significant motions, occlusions, and variations in brightness, rendering them prone to errors in complex regions.

**Rejection-based Methods.** Rejection methods, post the global registration procedure, discern and eliminate motion regions within the input data, followed by the fusion of static regions to reconstruct HDR images. Grosch et al. [3] devised an error map by assessing color disparities post alignment, aiming to exclude pixels with mismatches. Pece et al. [5] identified regions of motion through the utilization of a median threshold bitmap on input LDR images. Jacobs et al. [17] pinpointed areas of misalignment via an analytical approach involving weighted intensity variance analysis. These approaches often yield unsatisfactory HDR outcomes as they incur the loss of valuable information while eliminating pixels.

**Patch-based Methods.** The patch-based methods, involving patch-wise alignment among exposure images for deghosting, have been explored in the literature. Sen et al. [7] introduced a patch-based energy minimization method that simultaneously optimizes alignment and reconstruction. In the work by Hu et al. [6], an iterative propagation of intensity and gradient information was conducted using a coarse-to-fine schedule. Ma et al. [18] proposed an approach based on structural patch decomposition, which dissects an image patch into signal strength, signal structure, and mean intensity components for the reconstruction of ghost-free images. However, it is noteworthy that these methods lack compensation for saturation and are burdened by elevated computational costs.

**CNN-based Methods.** Kalantari et al. [8] initiated the alignment of images using optical flow and subsequently employed a CNN network for their fusion. Yan et al. [10] introduced a spatial attention mechanism based on CNN to mitigate issues related to motion and oversaturated regions. Yan et al. [19] formulated a non-local module aimed at expanding the receptive field for comprehensive global merging. In their work, Song et al. [20] harnessed the benefits of the Transformer’s extensive receptive field to globally recover areas affected by motion. Additionally, HyHDR [21] proposed an innovative patch aggregation module grounded in deep learning, strategically fusing valuable information from non-reference frames. Despite the significant performance breakthroughs achieved by these methodologies, their outcomes in both dynamic and static areas remain somewhat unsatisfactory.

### 2.2. Vision Transformer

Transformers have demonstrated remarkable success in natural language processing. The multi-head self-attention mechanism utilized in this context effectively captures long-range correlations among word token embeddings. A recent development, Vision Transformer (ViT) [22], has illustrated that a pure Transformer architecture can be directly applied to sequences of non-overlapping image patches, exhibiting excellent performance in image classification tasks. This showcases the versatility of Transformer models beyond natural language applications, extending their efficacy to the domain of computer vision. CA-ViT, proposed by Liu et al. [13], leverages the Transformer’s capability for capturing long-range dependencies and extracting global feature information, complementing it with the ability of CNN to extract local information. The collaboration between these features has proven to be highly effective. Building upon the Transformer architecture, Zamir et al. [23] introduced improvements by incorporating a locally aware Transformer design. This design enhances the model’s perception of local image details by introducing local convolution operations within the Transformer. Our approach is inspired by [13,23], strategically handling local and global information differently based on their inherent characteristics.

## 3. Method

In a series of LDR images with varying exposure levels, images of the same scene are grouped together. Each group comprises three images: underexposed, normally exposed, and overexposed. Our goal is to fuse the information from these three LDR images to reconstruct an HDR image without ghosting artifacts. In previous research [10,24], the authors utilized a set of three LDR images as input, designating the normally exposed image as the reference frame. Using the input images $\left\{L_{1},L_{2},L_{3}\right\}$, our model derives the HDR image $\hat{H}$ as follows:

(1) $$\hat{H}=f\left(L_{1},L_{2},L_{3};\theta \right),$$

where f(·) represents the HDR imaging function, and θ refers to the network’s parameters.

### 3.1. Overview of the HL-HDR Architecture

**Figure 1:**
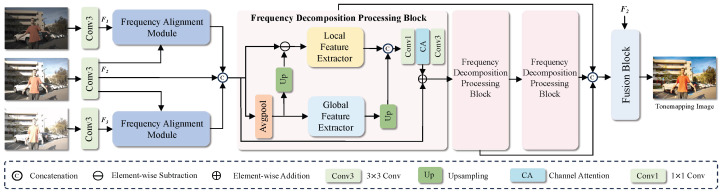
The HL-HDR framework consists of two main stages. First, the cross-frame feature alignment stage uses the Frequency Alignment Module to align overexposed and underexposed images to the reference frame. Second, the feature fusion stage stacks multiple Frequency Decomposition Processing Blocks to extract and integrate features, reconstructing high-quality, ghost-free HDR images.

As shown in Figure 1, the proposed HL-HDR framework consists of two main stages:

The first stage is the cross-frame alignment stage, corresponding to the Frequency Alignment Module in the figure. This module takes three images with different exposures as input and extracts shallow features through a shared-weight convolution, producing 64-channel feature maps $\left\{F_{1},F_{2},F_{3}\right\}$. The feature map of the normally exposed image, $F_{2}$, is used as the reference frame, while the underexposed feature map $F_{1}$ and overexposed feature map $F_{3}$ are aligned to it.

The second stage is the feature fusion stage, in which multiple Frequency Decomposition Processing Blocks are stacked to extract and integrate the aligned features. Specifically, the features are decomposed into high-frequency and low-frequency components and processed according to their characteristics: the high-frequency branch employs a Local Feature Extractor to capture local details through stacked convolutional blocks and enhances cross-frame high-frequency information via dense connections; the low-frequency branch uses a Global Feature Extractor to model long-range dependencies through multi-layer channel attention. The extracted high- and low-frequency features are then fused.

In the final reconstruction stage, the network reduces the number of channels while introducing long-range residual connections, ultimately reconstructing the output as a 3-channel HDR image.

### 3.2. Frequency Alignment Module

To balance large-scale motion modeling and detail preservation during the alignment stage, we first decompose both the reference frame and the non-reference frames into high-frequency and low-frequency components, which are then independently aligned in separate branches. Specifically, the low-frequency components are obtained by applying average pooling to the original feature maps, while the high-frequency components are derived by subtracting the low-frequency part from the original features. This process can be formally expressed as follows:

(2) $$f_{\mathrm{il}}=\mathrm{Avgpool}\left(F_{i}\right),$$

(3) $$f^{\prime }=\mathrm{Up}\left(f_{\mathrm{il}}\right),f_{\mathrm{ih}}=F_{i}-f^{\prime },$$

(4) $$f_{2l}=\mathrm{Avgpool}\left(F_{2}\right),$$

(5) $$f^{\prime \prime }=\mathrm{Up}\left(f_{2l}\right),f_{2h}=F_{2}-f^{\prime \prime },$$

where Avgpool(·) refers to average pooling, Up(·) stands for bilinear interpolation upsampling. $F_{i}$ refers to the non-reference frame, while $F_{2}$ refers to the reference frame.

For the alignment of high-frequency components, the high-frequency features of the reference frame and non-reference frames are concatenated and fed into a convolution-based attention module to generate an attention map, which is subsequently applied to the non-reference frame features. This module is similar to the implicit alignment mechanism in AHDR [10], guiding the network to focus on critical information across different exposures. Under the guidance of attention, the model can adaptively reweight the importance of different regions, thereby refining edges and textures, enhancing local detail restoration, and effectively suppressing ghosting while ensuring structural consistency. Formally, the process can be written as:

(6) $$F_{\mathrm{am}}=\mathrm{AM}\left(\mathrm{Concat}\left(f_{\mathrm{ih}},f_{2h}\right)\right),$$

(7) $$F_{h}=f_{\mathrm{ih}}\cdot F_{\mathrm{am}},$$

where AM(·) refers to the attention module, and (·) denotes element-wise multiplication.

**Figure 2:**
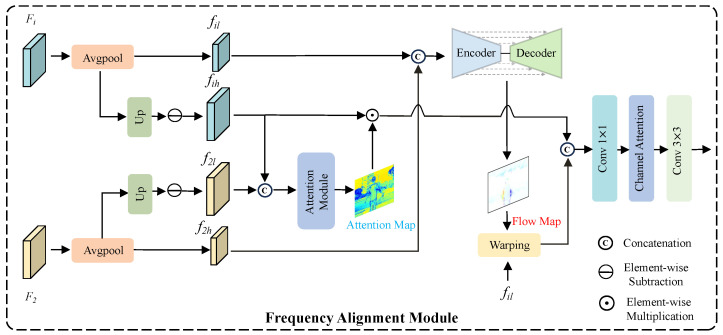
The architecture of the proposed FAM decomposes both the non-reference frames and the reference frame into high-frequency and low-frequency components, which are then aligned separately.

For the alignment of low-frequency components, we modify the Encoder-Decoder structure of SAFNet [11] to predict the optical flow field, which is then used to warp the low-frequency components of the non-reference frames, enabling more accurate modeling of large-scale motion. The above operations can be formulated as:

(8) $$F_{\mathrm{am}}=\mathrm{FM}\left(f_{\mathrm{il}},f_{2l}\right),$$

(9) $$F_{l}=\mathrm{Warp}\left(f_{\mathrm{il}},F_{\mathrm{am}}\right),$$

where FM(·) refers to the Encoder-Decoder structure shown in Figure 2, and Warp(·) denotes the warping operation.

Finally, the aligned low-frequency and high-frequency components are fused, followed by convolution and a channel attention module to restore channel information and further extract features. This process can be formally expressed as follows:

(10) $$F_{\mathrm{out}}=\mathrm{Conv}3(\mathrm{CA}\left(\mathrm{Conv}1\left(\mathrm{fusion}\left(\mathrm{Concat}\left(F_{l},F_{h}\right)\right)\right)\right),$$

where fusion(·) denotes the operation for integrating features at different scales, CA(·) represents channel attention, Conv1(·) stands for a 1×1 convolution, and Conv3(·) denotes a 3×3 convolution.

### 3.3. Frequency Decomposition Processing Block

**Figure 3:**
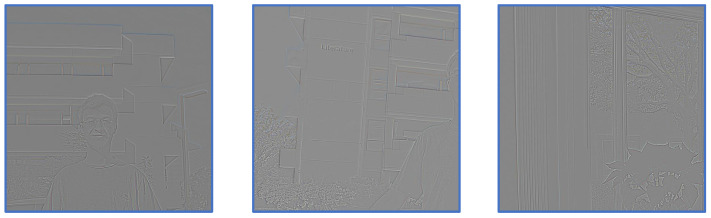
Visualization of the high-frequency components from three examples. Distinct structural edges and fine detail lines can be clearly observed, indicating that the use of the AvgPool operation effectively and accurately separates the high-frequency information.

To more clearly illustrate the high-frequency information, we present three representative examples in Figure 3. The visualizations demonstrate that structural edges and fine-grained details are effectively captured, confirming the reliability of the “average pooling + subtraction” operation for separating high- and low-frequency information. Motivated by this observation, we adopt a similar frequency decomposition strategy during the feature fusion stage to further enhance the network’s representational capacity. As shown in Figure 1, similar to the alignment stage, we also decompose the feature maps into high-frequency and low-frequency components during the feature fusion stage. By supplementing high-frequency details while extracting the global and background information of the image, this approach enhances local textures and edges, resulting in improved visual quality. This process can be formally expressed as follows:

(11) $$F_{\mathrm{low}}=\mathrm{Avgpool}\left(F\right),$$

(12) $$F^{\prime }=\mathrm{Up}\left(F_{\mathrm{low}}\right),F_{\mathrm{high}}=F-F^{\prime },$$

(13) $$F_{\mathrm{out}}=F+\mathrm{Conv}3(\mathrm{CA}\left(\mathrm{Conv}1\left(\mathrm{Concat}\left(\mathrm{LFE}\left(F_{\mathrm{high}}\right),\mathrm{GFE}\left(F_{\mathrm{low}}\right)\right)\right)\right),$$

where Avgpool(·) refers to average pooling, Up(·) stands for bilinear interpolation upsampling, LFE(·) stands for Local Feature Extractor, GFE(·) stands for Global Feature Extractor, CA(·) denotes channel attention, Conv3(·) stands for 3 × 3 convolution, and Conv1(·) stands for 1 × 1 convolution that restores the channel count from 128 to 64.

#### 3.3.1. Local Feature Extractor

**Figure 4:**
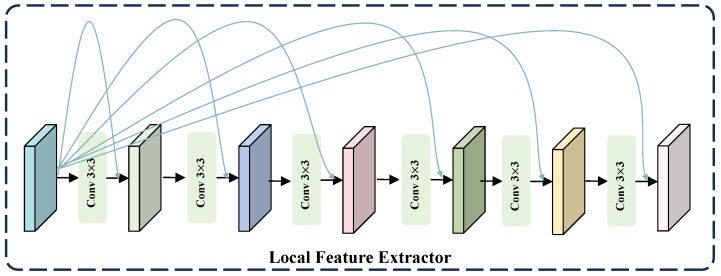
The architecture of the proposed LFE is comprised of a series of standard convolutions and dense residual connections.

To better recover the detailed information in an image, we process the high-frequency information, which inherently contains a wealth of detail. High-frequency information requires local details; thus, the use of convolutions with small kernels allows for a more focused extraction of these details. Moreover, given the superior capability of standard residual learning in maintaining stable feature propagation and enhancing high-frequency detail representation [25,26,27], we incorporate dense residual connections into the high-frequency information extraction process to fully leverage multi-level features and strengthen high-frequency detail modeling. Overall, we utilize six 3 × 3 convolutions. As depicted in Figure 4, we only display a portion of the residual connections, but in reality, these are dense residual connections. They are not merely connections between adjacent layers, but rather, the output of each layer is merged with the outputs of all preceding layers, enabling each layer to directly access the feature information of all previous layers.

#### 3.3.2. Global Feature Extractor

**Figure 5:**
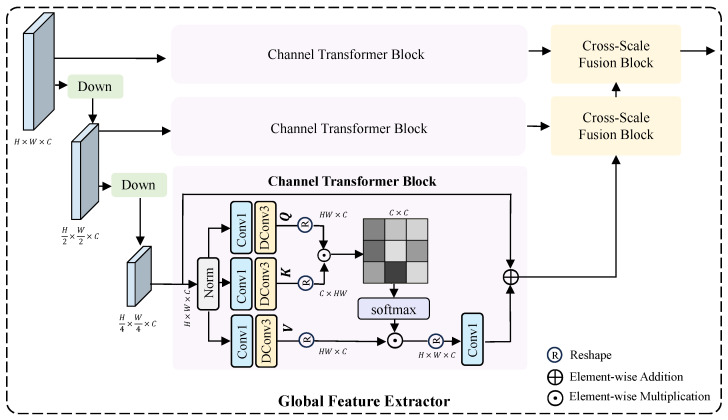
The architecture of the proposed GFE is designed for extracting low-frequency features.

For low-frequency information, it is necessary to leverage global context to restore the overall structure and background of the image. As shown in Figure 5, although multi-scale feature extraction enables long-range information interactions, some information may be lost during the downsampling process. To address this, we perform feature extraction at each scale to compensate for the information loss caused by downsampling. In each feature extraction layer, we introduce a Channel Transformer Block, which can establish global contextual information and possesses a global receptive field, making it highly suitable for extracting low-frequency features that depend on global information. Furthermore, to compensate for potential information loss when directly upsampling feature maps of different sizes and concatenating them, we employ the CSFM to effectively merge feature maps of varying resolutions.

**Channel Transformer Block.** Given that the channel-wise self-attention mechanism proposed in [23] can effectively model cross-channel dependencies while reducing computational complexity, our Transformer architecture abandons spatial self-attention and instead adopts channel-wise self-attention to achieve more efficient feature modeling. The input $X\in R^{H\times W\times C}$ is first layer-normalized to obtain a tensor $Y\in R^{H\times W\times C}$. Then, 1 × 1 convolutions are applied to aggregate pixel-wise cross-channel context, followed by 3 × 3 depth-wise convolutions to encode channel-wise spatial context. This process generates the query (Q), key (K), and value (V), which can be expressed mathematically as:

(14) $$Q=W_{p}^{Q}W_{d}^{Q}Y,\;K=W_{p}^{K}W_{d}^{K}Y,\;V=W_{p}^{V}W_{d}^{V}Y,$$

where $W_{p}\left(\cdot \right)$ represents the 1 × 1 point-wise convolution and $W_{d}\left(\cdot \right)$ represents the 3 × 3 depth-wise convolution.

Then reshape Q into $R^{HW\times C}$, reshape K into $R^{C\times HW}$. After this transformation, matrix multiplication can be performed, followed by a softmax operation to obtain an attention map $A\in R^{C\times C}$. Reshape V into $R^{HW\times C}$, allowing for matrix multiplication with A. The resulting output is reshaped into $R^{H\times W\times C}$, and finally, a residual connection is added by summing the initial feature map with the obtained feature map. The specific process is illustrated as follows:

(15) Attention(Q,K,V)=V·Softmax(Q·K/α),

(16) $$\hat{X}=Attention\left(\hat{Q},\hat{K},\hat{V}\right)+X,$$

where α is a learnable scaling parameter, X refers to the initial input feature map, and $\hat{X}$ refers to the final result.

Next, we utilize 1 × 1 Convolution to aggregate information from different channels and employ 3 × 3 depth-wise Convolution to aggregate information from spatially neighboring pixels. Additionally, we incorporate a gating mechanism to enhance information encoding. Finally, a long-range residual connection is added, summing the initial feature map with the feature map obtained at this stage.

**Figure 6:**
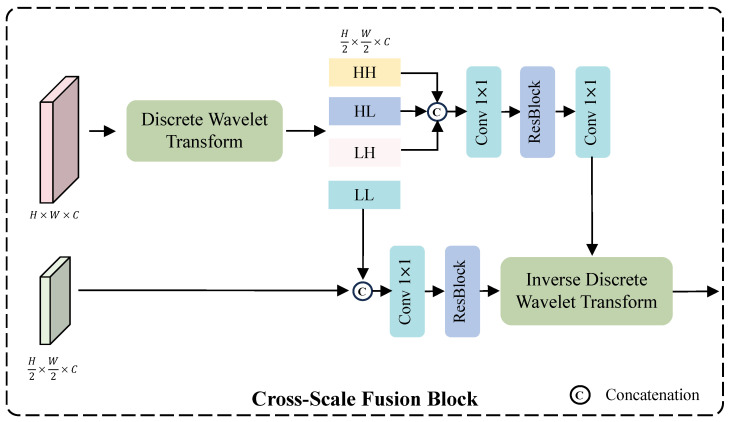
The architecture of the proposed CSFM. CSFM is a cross-scale fusion module that leverages wavelet transforms to effectively merge feature maps of different spatial resolutions.

**Cross-Scale Fusion Module.** Due to the differing spatial resolutions of features at various scales, they are typically adjusted to a unified resolution via downsampling or upsampling for feature fusion. However, such operations may lead to the loss of important structural details, thereby affecting the final image restoration. To alleviate this problem, we introduce a wavelet-based cross-scale feature fusion strategy that fully leverages the capability of wavelet transforms in representing multi-scale image structures [28]. As shown in Figure 6, we employ the Haar Discrete Wavelet Transform (DWT) to decompose a feature map $F_{b}\in R^{H\times W\times C}$ into four sub-bands: LL (Low–Low), LH (Low–High), HL (High–Low), and HH (High–High). Here, “L” and “H” denote low-pass and high-pass filtering along the horizontal and vertical dimensions, respectively. Each sub-band has half the spatial resolution of the original feature map, while the number of channels remains unchanged. Among these, the LL sub-band retains the low-frequency components, representing the global structure and smooth regions of the feature. It is concatenated with the small-scale feature map $F_{s}\in R^{\frac{H}{2}\times \frac{W}{2}\times C}$, followed by a 1×1 convolution for channel reduction (from 128 to 64) and a residual block for feature refinement. The HH, HL, and LH sub-bands preserve the high-frequency components, containing texture and edge details. After concatenation, a 1×1 convolution reduces the channels to 64, followed by a residual block for detailed feature extraction, and another 1×1 convolution restores the channel number to 192. The use of two 1×1 convolutions effectively reduces the number of parameters and computational complexity, since directly processing a 192-channel feature map would be computationally expensive. Finally, the refined high- and low-frequency features are recombined through the Inverse Discrete Wavelet Transform (IDWT) to produce the fused representation. This process can be formally expressed as follows:

(17) $$HH,HL,LH,LL=\mathrm{DWT}(F_{b}),$$

(18) $$f_{b}=\mathrm{Conv}1\left(\mathrm{Res}\left(\mathrm{Conv}1\left(\mathrm{Concat}\left(HL,LH,LL\right)\right)\right)\right),$$

(19) $$f_{s}=\mathrm{Conv}1\left(\mathrm{Res}\left(\mathrm{Concat}\left(F_{s},LL\right)\right)\right),$$

(20) $$F=\mathrm{IDWT}(f_{b},f_{s}),$$

where Res(·) represents the residual block.

### 3.4. Training Loss

Due to the typical display of HDR images after tonemapping, training the network on tonemapped images is more effective than training directly in the HDR domain. When provided with an HDR image H in the HDR domain, we compress the image’s range using the μ-law transformation.

(21) $$T\left(H\right)=\frac{\log(1+\mu H)}{\log(1+\mu )},$$

where μ represents a parameter that defines the degree of compression, and T(H) represents the tonemapped image. Throughout this work, we maintain *H* within the range [0, 1] and set μ to 5000.

$\hat{H}$ is the predicted result obtained from our HL-HDR model, and H is the Ground Truth. Here, we employ $L_{1}$ loss to compute the loss. Additionally, we use an auxiliary perceptual loss $L_{p}$ for supervision [13]. The perceptual loss measures the difference between the output image and the Ground Truth image in the feature representations of multiple layers in a pre-trained CNN, achieved by computing the mean squared error between the feature maps of each layer. We can express this as follows:

(22) $$L_{1}={∥T\left(H\right)-T\left(\hat{H}\right)∥}_{1},$$

(23) $$L_{p}={∥\theta_{j}\left(T\left(H\right)\right)-\theta_{j}\left(T\left(\hat{H}\right)\right)∥}_{1},$$

where $\theta_{j}$ represents the $j^{th}$ convolutional feature extracted from the pre-trained VGG-16 network, with *j* denoting the $j^{th}$ layer.

Therefore, our final loss function is the result of adding $L_{1}$ and $L_{p}$, with different weights assigned to each, for which we introduce a parameter λ. The final loss function can be expressed by the following formula:

(24) $$L_{total}=L_{1}+\lambda L_{p},$$

where λ is set to 0.01.

## 4. Experiments

### 4.1. Experiments Settings

**Datasets.** The proposed method has been trained on three distinct datasets: Kalantari’s dataset [8], Tel’s dataset [14], and Hu’s dataset [29]. Kalantari’s dataset consists of 74 training samples and 15 testing samples captured from real-world scenes, with exposure values set at {−2, 0, +2} and {−3, 0, +3}. Tel’s dataset comprises 108 training samples and 36 testing samples. For Hu’s dataset, the first 85 samples were used for training, while the remaining 15 were reserved for testing. This dataset employs an exposure bias of {−2, 0, +2} and is synthetically generated using a game engine sensor. To evaluate the effectiveness and generalization capability of the proposed model, we conducted tests on Sen’s dataset [7] and Tursun’s dataset [30] using weights pre-trained on Kalantari’s dataset. Since these two datasets contain only LDR images at different exposure levels and lack ground truth, the performance comparison across methods is limited to subjective assessment.

**Evaluation Metrics.** We use five objective measures for quantitative comparison: PSNR-μ, SSIM-μ, PSNR-*l*, SSIM-*l*, and HDR-VDP-2 [31], where μ and *l* indicate that the metrics are computed in the tonemapped domain and the linear domain, respectively. Among these, HDR-VDP-2 is a perceptual quality metric specifically designed for HDR images. It models the human visual system’s sensitivity to luminance, contrast, and local structural variations, providing a more accurate assessment of perceived image distortions compared to traditional pixel-wise metrics.

**Implementation Details.** Our implementation is based on PyTorch 3.9. Before training, we sample 256×256 patches from the dataset with a stride of 64. To enhance the diversity of the training data, we apply data augmentation techniques including rotation and flipping, as well as their combinations, resulting in six different augmentation strategies. We employ the Adam optimizer with a batch size of 8 and an initial learning rate of $2\times 10^{-4}$, which is reduced every 70 epochs. The model is trained for a total of 250 epochs on a single NVIDIA GeForce RTX 4090 GPU (NVIDIA, Santa Clara, CA, USA).

### 4.2. Comparison with the State-of-the-Art Methods

To comprehensively evaluate the performance of our model, we compared it against representative state-of-the-art deep learning-based approaches spanning different architectural paradigms. Specifically, we considered six CNN-based methods, including DHDR [9], AHDR [10], NHDRR [19], APNT [32], PGN [33], and SAFNet [11]; one GAN-based method, HDR-GAN [34]; three Transformer-based models, namely CA-ViT [13], SCTNet [14], and HyHDR [21]; as well as two diffusion-based methods, DiffHDR [35] and LFDiff [12].

**Datasets with Ground Truth.**Table 1, Table 2 and Table 3 presents the quantitative results of HL-HDR on three datasets. Our method is compared against several state-of-the-art approaches using the testing data from [8,14,29], which consist of challenging samples characterized by saturated backgrounds and foreground motions. The average of all quantitative results is computed across the testing images.

Notably, our method performs remarkably well on Kalantari’s dataset [8], achieving state-of-the-art performance in PSNR-μ, along with competitive results in other metrics. On Hu’s dataset [29], our method achieves strong performance in both PSNR-μ and PSNR-*l*, with PSNR-*l* outperforming the second-best approach by 0.82 dB. On Tel’s dataset [14], our method demonstrates overall superiority, where both PSNR-μ and PSNR-*l* substantially surpass the second-best approach, with gains of 0.62 dB and 0.32 dB, respectively.

**Figure 7:**
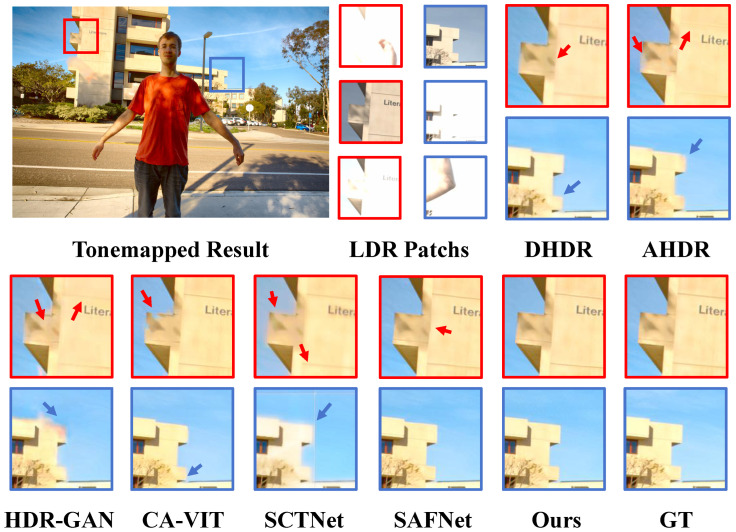
Examples of Kalantari et al.’s dataset [8].

**Figure 8:**
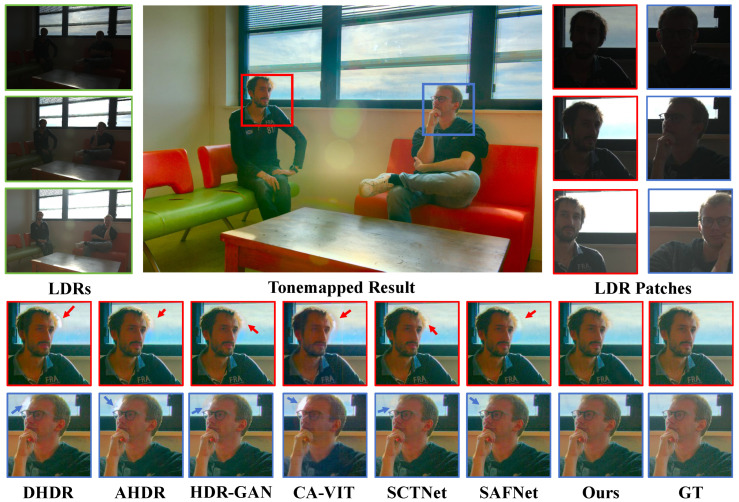
Examples of Tel et al.’s dataset [14].

**Figure 9:**
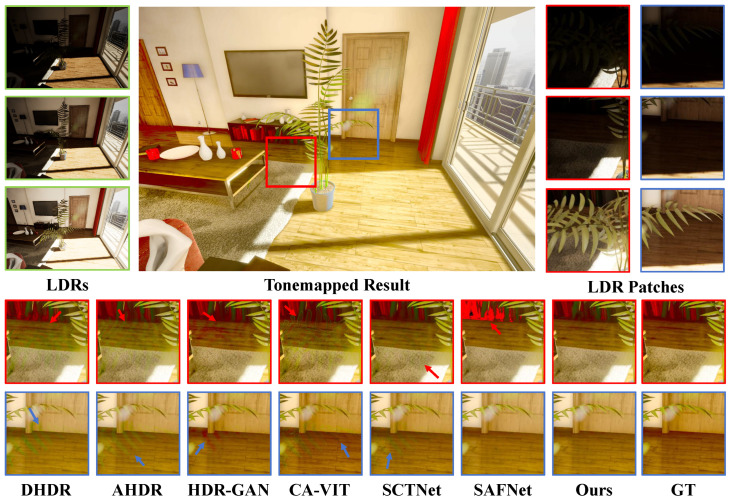
Examples of Hu et al.’s dataset [29].

In Figure 7, Figure 8 and Figure 9, the datasets present significant challenges due to large-scale foreground motion and severe over/under-exposed regions. We qualitatively compare our method with several state-of-the-art approaches. Most competing methods suffer from ghosting artifacts in regions with motion and saturation. On Kalantari’s dataset [8], DHDR [9] exhibits severe ghosting, while AHDR [10], HDR-GAN [34], and SCTNet [14] not only fail to recover complete structural information but also perform poorly in detail restoration. For example, in the patch comparison shown in Figure 7, these three methods fail to reconstruct the balcony, with sky elements incorrectly blended in, and the wall textures appear blurry. CA-VIT [13] and SCTNet [14] further suffer from blocky ghosting artifacts due to patch-based sampling. In contrast, SAFNet [11] exhibits only slight wall deformation. Our proposed method not only restores the overall structural content accurately but also excels in preserving fine details. In particular, the wall lines remain sharp and clear, demonstrating the strong capability of our model in capturing and restoring fine-grained information.

In Figure 8, we show a comparison scene from Tel’s dataset [14], where only the heads of two people exhibit slight motion. All other methods, however, produced noticeable ghosting artifacts in these motion regions. In contrast, our method accurately detects the motion areas and achieves superior image reconstruction. In Figure 9, we show a comparison scene from Hu’s dataset [29], where the motion is much more substantial. Except for our method, all other approaches generated large ghosting regions in the motion areas, significantly degrading the visual quality.

**Figure 10:**
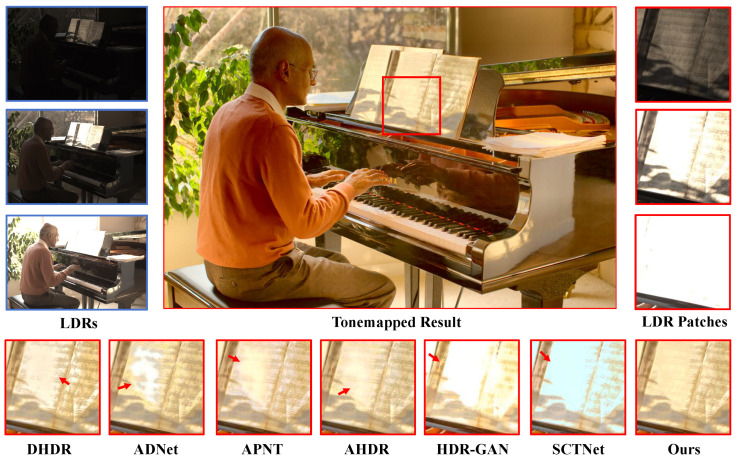
Example from Sen et al.’s dataset [7].

**Figure 11:**
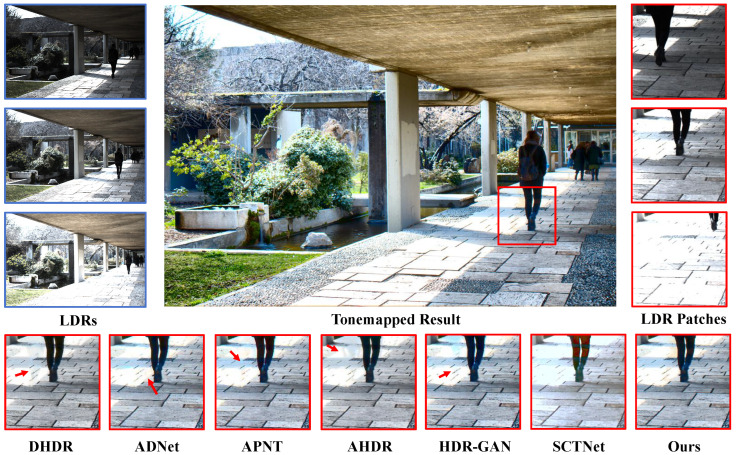
Example from Tursen et al.’s dataset [30].

**Evaluation on Datasets without Ground Truth.** To evaluate the generalization capability of the proposed HDR imaging method, we tested the model trained on Kalantari’s Dataset [8] on Sen’s Dataset [7] and Tursun’s Dataset [30], both of which lack ground truth. Consequently, the quality of the generated HDR images can only be assessed through subjective visual inspection. Notably, in Figure 10, most methods fail to recover overexposed regions, whereas our method performs exceptionally well, not only avoiding overexposure but also successfully restoring rich detail. In Figure 11, both our method and SCTNet are visually the most appealing, with no noticeable ghosting caused by human motion. This is because the scene contains abundant background information, and Transformer-based methods can fully exploit long-range dependencies to extract information from similar regions, thereby restoring details in motion areas and generating ghost-free images.

### 4.3. Computational Budgets

We further compared model parameters and inference times, as summarized in Table 4. Traditional patch match–based methods, such as Sen [7] and Hu [6], exhibit very long inference times due to their reliance on CPU computation. CA-ViT [13] employs standard Transformer blocks, which results in relatively high computational cost despite a moderate number of parameters. DiffHDR [35], reconstructing HDR images from pure noise, incurs both high inference time and a large parameter count. SAFNet [11] achieves fast inference with a small model size, but its reconstruction performance still leaves room for improvement. LFDiff [12], although faster than earlier diffusion-based models, still relies on the diffusion process, and our method achieves roughly three times its inference speed. In comparison, our method attains competitive inference speed (0.21 s) on a single A100 GPU with a moderate parameter count (4.08 M), striking a good balance between computational efficiency and model capacity, while also demonstrating superior reconstruction performance.

### 4.4. Ablation Studies

We conducted ablation experiments on the Kalantari dataset to evaluate the effectiveness of each module in our model. The following sections present the ablation analysis from three perspectives, corresponding to the main components of the model.

#### 4.4.1. Effect of Different Alignment Modules

To validate the effectiveness of the proposed Frequency Alignment Module, we compared it with two alternative models: one without any alignment module, and the other using the AHDR [10] alignment module. All other model components and parameters were kept identical.

**Figure 12:**
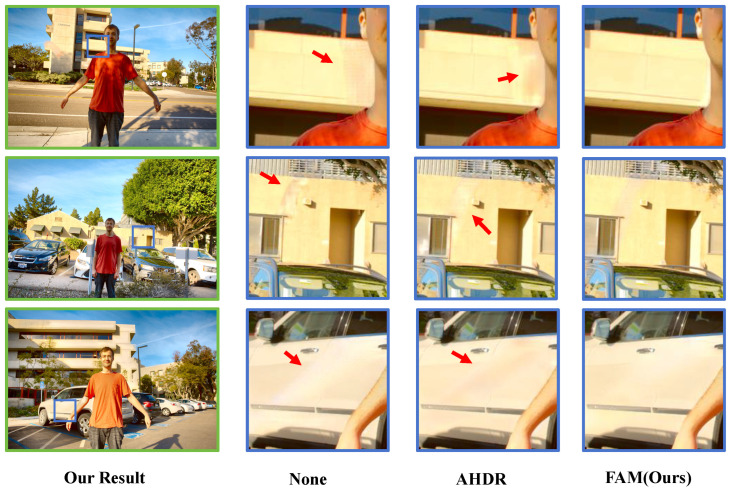
Comparison of different alignment strategies.

As shown in Table 5, it is evident that the model without any alignment module achieves the lowest metrics. The model using AHDR [10] for alignment shows a slight improvement, but the gain is limited. In contrast, when aligned using our proposed FAM, both PSNR-μ and PSNR-*l* increase significantly, with PSNR-*l* rising by 0.63 dB. Although the model without alignment achieves relatively lower scores, it still outperforms several existing methods such as CA-ViT [13] and SCTNet [14], indirectly highlighting the effectiveness of our proposed FDPB. In addition, we provide visual comparisons to further demonstrate the effectiveness of our alignment strategy. As shown in Figure 12, we select three representative examples from the test set that involve large-scale motion and overexposed regions. The model without alignment produces noticeable ghosting artifacts, while the AHDR-aligned model achieves partial improvement but still suffers from residual artifacts. In contrast, our FAM-aligned results almost completely eliminate ghosting. This superior performance can be attributed to the incorporation of optical flow within FAM, which effectively captures large-scale motion and further enhances the quality of HDR image reconstruction.

#### 4.4.2. Ablation Analysis of Components in the Frequency Alignment Module

To validate the rationale behind our proposed FAM, which decomposes high- and low-frequency features and processes them using different methods, we designed four experimental settings: (1) No high–low-frequency separation, aligning the two frames using only optical flow; (2) No high–low-frequency separation, aligning the two frames using convolution and attention(AHDR-aligned); (3) High–low-frequency separation, aligning both high- and low-frequency features using optical flow; (4) High–low-frequency separation, aligning both high- and low-frequency features using convolution and attention.

Table 6 presents the comparison results of different alignment strategies for high- and low-frequency features. As shown in the table, the non-separation strategies (1) and (2) exhibit notable differences: the optical-flow-based approach achieves significantly better PSNR-*l* and SSIM-*l* compared to the convolution + attention approach, demonstrating the superiority of optical flow in handling large-scale motion. When high- and low-frequency features are separated and both aligned using optical flow (3), the performance is comparable to (1) but with only marginal improvement, indicating that separation alone does not yield substantial benefits. In contrast, fully relying on convolution and attention after separation (4) performs even worse than the non-separated cases, highlighting its limitations in capturing large-scale motion. In comparison, our proposed FAM achieves the best results in PSNR-μ, SSIM-μ, and SSIM-*l*, while maintaining overall stable performance. These results validate the effectiveness of combining optical flow with frequency separation in our design.

#### 4.4.3. Ablation Analysis of Components in the Frequency Decomposition Processing Block

To validate the effectiveness of the proposed FDPB, we design a more fine-grained ablation study consisting of six comparative schemes: (1) Without frequency decomposition, the aligned features are simultaneously fed into both the Global Feature Extractor (GFE) and the Local Feature Extractor (LFE); (2) With frequency decomposition, but with swapped branch functions, where the high-frequency features are processed by the Global Feature Extractor (GFE) and the low-frequency features are processed by the Local Feature Extractor (LFE); (3) With frequency decomposition, but extracting both high- and low-frequency features using the LFE; (4) With frequency decomposition, but extracting both high- and low-frequency features using the GFE; (5) Removing the dense residual connections in the low-frequency branch; (6) Removing the wavelet-based Cross-Scale Fusion Module at each layer of the high-frequency branch.

Table 7 presents a comprehensive ablation study of the proposed Frequency Decomposition Processing Block (FDPB). It can be observed that performing frequency decomposition significantly enhances HDR reconstruction performance: comparing the scheme without decomposition (1) to the schemes with decomposition (3) and (4), PSNR-*l* is generally improved, indicating that separating high- and low-frequency features facilitates more effective extraction of global structures and local details. Notably, although scheme (4) achieves the highest PSNR-*l*, its PSNR-μ is relatively low due to insufficient processing of high-frequency information, demonstrating that relying solely on the Global Feature Extractor (GFE) is inadequate for restoring image details. The importance of matching feature types to the appropriate extractor is highlighted in scheme (2), where swapping the high-frequency and low-frequency branches leads to a noticeable performance drop, showing that high-frequency features are better handled by the Local Feature Extractor (LFE) and low-frequency features by the GFE. Furthermore, removing dense residual connections in the low-frequency branch (5) or the Cross-Scale Fusion Module in the high-frequency branch (6) results in decreased performance, emphasizing the critical role of these components in preserving structural information and enhancing details. Overall, the complete FDPB design, integrating frequency decomposition, proper branch assignment, dense residual connections, and wavelet-based cross-scale fusion, achieves the best results across all metrics, confirming its effectiveness in restoring high-quality, ghost-free HDR images.

## 5. Conclusions

This paper presents HL-HDR, a high–low-frequency-aware HDR reconstruction network for dynamic scenes. It explicitly decomposes features into high- and low-frequency components, effectively combining global context modeling with fine detail restoration. The Frequency Alignment Module (FAM) enables precise motion estimation and structure preservation, while the Frequency Decomposition Processing Block (FDPB) supports hierarchical cross-scale feature fusion. Compared with state-of-the-art models such as LFDiff and SAFNet, HL-HDR offers clear advantages. Unlike diffusion-based models like LFDiff, which rely on iterative sampling and are computationally expensive, HL-HDR has a compact architecture, stable training, and faster convergence. Unlike lightweight CNNs such as SAFNet, which often sacrifice detail for speed, HL-HDR jointly optimizes high- and low-frequency features to balance visual quality and efficiency. Experiments on multiple public HDR benchmarks show that HL-HDR consistently improves performance. On three datasets with ground-truth HDR images, PSNR-μ increases by 0.05 dB, 0.62 dB, and 0.28 dB, with visual results showing better ghost removal and detail preservation. For future work, we plan to explore lightweight designs and real-time deployment, leverage diffusion models to improve generalization and robustness in complex dynamic scenes, and build larger multi-scene HDR datasets to promote practical applications.

## Figures and Tables

**Table 1:** Quantitative comparisons on Kalantari’s dataset [8]. The best results are highlighted in red, and the second-best results are highlighted in blue.

Methods	PSNR-μ	PSNR-*l*	SSIM-μ	SSIM-*l*	HDR-VDP-2
DHDR [9]	41.64	40.91	0.9869	0.9858	60.50
AHDR [10]	43.62	41.03	0.9900	0.9862	62.30
NHDRR [19]	42.41	41.08	0.9887	0.9861	61.21
HDR-GAN [34]	43.92	41.57	0.9905	0.9865	65.45
APNT [32]	43.94	41.61	0.9898	0.9879	64.05
CA-ViT [13]	44.32	42.18	0.9916	0.9884	66.03
HyHDR [21]	44.64	42.47	0.9915	0.9894	66.05
DiffHDR [35]	44.11	41.73	0.9911	0.9885	65.52
SCTNet [14]	44.43	42.21	0.9918	0.9891	66.64
PGN [33]	44.73	42.27	0.9918	0.9890	66.08
SAFNet [11]	44.66	43.18	0.9919	0.9901	66.11
LFDiff [12]	44.76	42.59	0.9919	0.9906	66.54
Ours	44.81	42.69	0.9921	0.9901	66.71

**Table 2:** Quantitative comparisons on Tel’s dataset [14]. The best results are highlighted in red, and the second-best results are highlighted in blue.

Methods	PSNR-μ	PSNR-*l*	SSIM-μ	SSIM-*l*	HDR-VDP-2
DHDR [9]	40.05	43.37	0.9794	0.9924	67.09
AHDR [10]	42.08	45.30	0.9837	0.9943	68.80
NHDRR [19]	36.68	39.61	0.9590	0.9853	65.41
HDR-GAN [34]	41.71	44.87	0.9832	0.9949	69.57
CA-ViT [13]	42.39	46.35	0.9844	0.9948	69.23
SCTNet [14]	42.55	47.51	0.9850	0.9952	70.66
DiffHDR [35]	42.18	45.63	0.9841	0.9946	69.88
SAFNet [11]	42.68	47.46	0.9792	0.9955	68.16
Ours	43.30	47.83	0.9878	0.9957	70.73

**Table 3:** Quantitative comparisons on Hu’s dataset [29]. The best results are highlighted in red, and the second-best results are highlighted in blue.

Methods	PSNR-μ	PSNR-*l*	SSIM-μ	SSIM-*l*	HDR-VDP-2
DHDR [9]	41.13	41.20	0.9870	0.9941	70.82
AHDR [10]	45.76	49.22	0.9956	0.9980	75.04
NHDRR [19]	45.15	48.75	0.9956	0.9981	74.86
HDR-GAN [34]	45.86	49.14	0.9945	0.9989	75.19
APNT [32]	46.41	47.97	0.9953	0.9986	73.06
CA-ViT [13]	48.10	51.17	0.9947	0.9989	77.12
HyHDR [21]	48.46	51.91	0.9959	0.9991	77.24
DiffHDR [35]	48.03	50.23	0.9954	0.9989	76.22
SCTNet [14]	48.10	51.03	0.9963	0.9991	77.14
PGN [33]	48.66	52.49	0.9965	0.9992	77.33
SAFNet [11]	47.18	49.35	0.9951	0.9990	76.83
LFDiff [12]	48.74	52.10	0.9968	0.9993	77.35
Ours	49.02	52.92	0.9970	0.9992	77.55

**Table 4:** Average runtime performance of various methods on the testing set of Kalantari’s dataset [8]. The inference times are measured on a single A100 GPU under a resolution of 1500×1000.

**Methods**	Sen [7]	Hu [6]	AHDR [10]	CA-ViT [13]	DiffHDR [35]	SAFNet [11]	LFDiff [12]	**Ours**
**Environment**	(CPU)	(CPU)	(GPU)	(GPU)	(GPU)	(GPU)	(GPU)	(GPU)
**Times (s)**	61.81 s	79.77 s	0.30 s	5.34 s	7.53 s	0.13 s	0.72 s	0.21 s
**Para. (M)**	–	–	1.24 M	1.22 M	74.99 M	1.12 M	7.48 M	4.08 M

**Table 5:** Comparison of different alignment modules. The best results are highlighted in red.

Alignment Module	PSNR-μ	PSNR-*l*	SSIM-μ	SSIM-*l*	HDR-VDP-2
None	44.53	42.06	0.9917	0.9890	66.24
AHDR	44.62	42.22	0.9919	0.9892	66.37
**FAM (Ours)**	44.81	42.69	0.9921	0.9906	66.71

**Table 6:** Comparison of different alignment strategies for high- and low-frequency features. The best results are highlighted in red.

Method	PSNR-μ	PSNR-*l*	SSIM-μ	SSIM-*l*	HDR-VDP-2
(1) No separation, optical flow	44.67	42.81	0.9920	0.9904	66.51
(2) No separation, conv + attention	44.62	42.22	0.9919	0.9892	66.43
(3) Separation, optical flow	44.65	42.67	0.9920	0.9896	66.49
(4) Separation, conv + attention	44.51	42.14	0.9920	0.9898	66.55
**FAM (Ours)**	44.81	42.69	0.9921	0.9906	66.71

**Table 7:** Ablation study of the proposed FDPB. The best results are highlighted in red.

Method	PSNR-μ	PSNR-*l*	SSIM-μ	SSIM-*l*	HDR-VDP-2
(1) No decomposition, GFE+LFE	44.49	42.61	0.9919	0.9896	66.37
(2) Frequency decomposition, swapped	44.45	42.33	0.9918	0.9895	66.14
(3) Frequency decomposition, LFE only	44.36	42.22	0.9918	0.9892	66.06
(4) Frequency decomposition, GFE only	44.64	42.88	0.9920	0.9900	66.64
(5) Low-freq w/o dense res.	44.68	42.59	0.9920	0.9902	66.61
(6) High-freq w/o CSFM	44.61	42.56	0.9919	0.9901	66.48
**FDPB (Ours)**	44.81	42.69	0.9921	0.9906	66.71

## Data Availability

The code is publicly available at https://github.com/chengeng0613/HL-HDR_Plus.
